# Sentinel lymph-node biopsy in head and neck cancer

**DOI:** 10.1038/sj.bjc.6601877

**Published:** 2004-06-08

**Authors:** S Höft, S Maune, C Muhle, W Brenner, N Czech, W-U Kampen, U Jänig, M Laudien, S Gottschlich, P Ambrosch

**Affiliations:** 1 Department of Otorhinolaryngology, Head and Neck Surgery, University Hospital of Schleswig-Holstein, Campus Kiel, Arnold-Heller-Str., 24105 Kiel, Germany; 2 Clinic of Nuclear Medicine, University Hospital of Schleswig-Holstein, Campus Kiel, Arnold-Heller-Str., 24105 Kiel, Germany; 3 Institute of Paidopathology, University Hospital of Schleswig-Holstein, Campus Kiel, Michaelisstr. 24105 Kiel, Germany

**Keywords:** sentinel lymph-node biopsy, lymphatic metastasis, head and neck neoplasms

## Abstract

The aim of the study was to assess the diagnostic value of the sentinel node method in patients suffering from squamous cell carcinoma of the upper aerodigestive tract. In 50 patients with oral, pharyngeal or laryngeal carcinomas staged N0 up to 50 MBq technetium-99m colloid were injected peritumorally. Sentinel nodes were localised using a *γ*-probe in the setting of an elective neck dissection. Pathological findings of sentinel nodes and corresponding neck specimens were compared. In 46 patients sentinel nodes were detected. Of these 34 patients were free of metastatic disease in the sentinel nodes and in the neck specimens. In 12 patients clinically occult metastases were found in the sentinel nodes. Three metastases were detected only after additional sectioning of the sentinel nodes. In four patients, a sentinel lymph node could not be localised. Our results support the sentinel node concept in head and neck cancer and a definition of the sentinel nodes as the three nodes with the highest activity. Careful clinical staging of the neck and thorough pathological evaluation of the sentinel nodes are necessary to avoid false-negative results.

Surgical therapy of the lymphatic basins in head and neck malignancies has been evolved from radical neck dissection to modified radical neck dissection in order to reduce the morbidity of the procedure and to preserve as much function and quality of life as possible for the patient while still maintaining an oncologic sound result (Suárez, 1962). With the same objective proponents of a further limitation favour a selective neck dissection in the presence of initial cervical disease (Traynor *et al*, 1996; Ambrosch *et al*, 2001). While there are efforts to reduce the surgical approach in the presence of metastases, there is even more reason to do so if a neck has been staged N0. The problem arises from the fact that current staging methods cannot reliably exclude small metastases. A risk of occult metastatic disease exceeding 15–20%, therefore, is considered an indication for elective neck dissection (Steiner and Hommerich, 1993; Pitman *et al*, 1997). If occult metastases are detected in the neck dissection specimen, adjuvant radiotherapy will usually be indicated while in case of a neck without metastatic disease there are no therapeutic consequences. However, patients experience a loss of quality of life due to the morbidity of the neck dissection. To preserve quality of life while aiming for an oncologic sound result, the sentinel lymph-node concept was established. Sentinel lymph nodes are defined as the first nodes to drain a tumour. Thus, they carry the highest risk of metastatic disease. Based on this apriority, the hypothesis of the sentinel node concept is that the oncologic status of the sentinel node has diagnostic value for the total lymphatic basin. The intriguing idea is that a sentinel node free of tumour may make an elective lymphonodectomy of the lymphatic basin unnecessary. The sentinel node concept has been introduced in malignant melanomas and breast cancer (Morton *et al*, 1992; Giuliano *et al*, 1994). While there have been early studies on malignant melanomas in the head and neck (Morton *et al*, 1993), there is only limited experience in head and neck squamous cell carcinoma. It was the aim of this study to assess the diagnostic value of the sentinel node method in head and neck cancer.

## PATIENTS AND METHODS

### Patients

From May 2000 until May 2003, 40 male and 10 female patients with previously untreated squamous cell carcinomas of the upper aerodigestive tract were included in this study. The study was conducted in accordance to the Revised Declaration of Helsinki (2000). Informed written consent was obtained from each patient. Tumours were located in the larynx in 12 cases. In all, 11 patients suffered from carcinoma of the tonsil, 10 patients of the mobile tongue, eight of the floor of the mouth, three of the base of the tongue, four of the palate, one of the dorsal oropharyngeal wall and one of the hypopharynx (Table 1

**Table 1:** Patient characteristics

				**ND**		**Number and level of SLNs**		**Number and level of SLNs with tumor**			**Level of metastases**	
**No.**	**Site of primary**	**Midline**	**T-status**	**Ipsi**	**Contra**	**Ipsi**	**Contra**	**Ipsi**	**Contra**	**pN-status**	**Ipsi**	**Contra**
1	Tongue	No	1	I–III	I-III	II, 8xIII	III			0		
2	Larynx	No	3	II–IV	II-IV	II	0			0		
3	Larynx	No	3	II–IV	II-IV	I, II, III	0			0		
4	Larynx	No	3	II-IV	II-IV	II, 2xIII, 2xV	0			0		
5	Tongue	No	2	I–III		4xIII		III		1	III	
6	Tonsil	No	2	I–IV	I-IV	3xI, 3xIII, 5xV	0	I		1	I	
7	Larynx	No	3	I–III		II				0		
8	Tongue	No	2	I–IV		II, 5xIII, IV, 1xIV				0		
9	Larynx	No	3	I–V	II-IV	I, II, 2xIII	0			0		
10	Tonsil	No	4	I–V		5xII				0		
11	Tonsil	No	2	I–V		I, 2xII				0		
12	FOM	No	2	I–III		I				0		
13	BOT	No	3	I–IV		II, V		II		1	II	
14	FOM	No	2	I–V		4xII				0		
15	Tongue	No	2	I–IV		II, III, V				0		
16	Larynx	No	1	I–IV		II, III				0		
17	Larynx	No	3	I–III	I-III	II	II			0		
18	Tonsil	No	2	I–IV		2xII		2xII		2b	2xII	
19	Tonsil	No	2	I–IV		II		II		2b	I, 2xII, III	
20	Tonsil	No	1	I–V		II				0		
21	Tongue	No	1	I–IV		4xII		II		1	II	
22	Tongue	No	2	I–IV		4xII, 2xIII		2xII		2b	2xII	
23	FOM	Yes	2	I–IV	I-IV	II	2xII, 3xIII			0		
24	Larynx	Yes	3	I-III	I-III	III	II, 2xIII			0		
25	Palate	Yes	2	II–III	II-III	III	II			0		
26	BOT	No	1	I–IV	I-IV	I, II, 2xIII	0			0		
27	Larynx	No	3	II–IV	II-IV	4xII	0			0		
28	Tongue	No	2	I–III		I, 2xIII				0		
29	Tongue	No	1	I–III		2xIII				0		
30	FOM	Yes	2	I–III	I–III	II	II			0		
31	FOM	Yes	3	I–IV	I–III	2xII	I, II			0		
32	BOT	No	3	II–IV	II–IV	2xII	0			0		
33	Tonsil	No	2	I–IV		II		II		2b	3xII	
34	Oropha	Yes	2	II–V	II–V	II	II			0		
35	FOM	No	2	I–IV	I–IV	I, II	0			0		
36	Larynx	No	1	II–IV		2xIII				0		
37	Palate	Yes	3	II–IV	II–IV	II	2xII		1	2c		II
38	Tonsil	No	2	I–IV		0				0		
39	Tongue	No	2	I–III		3xII, 3xIII		II, III		2b	II, III	
40	Tonsil	No	2	II–IV		II		II		1	II	
41	Tongue	No	2	I–IV		I, II, III		III		2b	2xI, III	
42	Tongue	No	3	I–III		II, III, IV				0		
43	Palate	No	2	II–III		0				0		
44	Tonsil	No	2	II–III		2xII				0		
45	FOM	No	1	I–III	I–II	I, II				0		
46	Hypopharynx	No	4	II–IV	II–IV		II			0		
47	Palate	No	2	II–III		0				1	II	
48	Larynx	Yes	3	II–III	II–III	II, 2xIII				0		
49	Larynx	No	3	II–III		0				0		
50	Tonsil	No	1	II–IV		II, 2xIII, IV				0		

Midline signifies a tumour crossing the midline. FOM=floor of the mouth; BOT=base of the tongue; oropha=posterior oropharyngeal wall; ND=levels of neck dissection; Ipsi=ipsilateral; Contra=contralateral; SLN=sentinel lymph node. Roman numbers indicate the levels of the neck.

). One more patient with a tumour of the base of the tongue and three more patients with laryngeal tumours who were all initially thought feasible for the study were not included because it was not possible to expose the caudal rim of the tumours and to perform a peritumoral injection of the tracer. Thus, the patients received only part of the injections.

Staging of the neck was based on ultrasound examination of the neck. In 19 patients, an ultrasound-guided fine-needle aspiration cytology was performed. In each case, the N0-status of the neck was confirmed as a prerequisite for study inclusion.

### Methods

Up to a total of 50 MBq ^99^Tc-colloid (Solco®Nanocoll, Solco, Basel, Switzerland) dissolved in 0.2 ml saline solution was injected peritumorally with a minimum of four injections depending on the location and size of the tumour. Particle size of the tracer was less than 80 nm in diameter. The injection was given on the day of resection of the primary tumour and elective neck dissection. In 11 patients with accessible tumours peritumoral injection was given preoperatively and lymphoscintigraphy was performed. Planar images were acquired using a large-field-of-view-gamma camera equipped with a LEAP-collimator (Gammadiagnost Tomo, Philips, Hamburg, Germany). The sequence for the first 10 min was 30 s per frame from a frontal, left or right lateral view. Then, 5-min images were acquired up to 30 min post injection from different views. Lymphatic drainage was assessed by visual inspection. Lymph nodes accumulating tracer were marked on the skin. Initially, only in cases with difficult to access tumours such as carcinomas of the base of the tongue, larynx and in some tumours of the oropharynx injection was performed intraoperatively. After May 2002, all tumours were injected intraoperatively. In these cases a lymphoscintigraphy to visualise the sentinel lymph node preoperatively was not possible. In patients with laryngeal tumours a microlaryngoscopy was performed to expose the tumour. Peritumoral injection was given by using a butterfly-cannula.

For intraoperative detection of the sentinel lymph nodes, cutaneous flaps were raised and the sternomastoid muscle was retracted. A straight 14-mm diameter *γ*-probe (Navigator GPS, RMD, Watertown, MA, USA) was used to localise lymph nodes accumulating tracer. Counts of the primary tumour, background activity and sentinel lymph nodes were documented for a 10-s-period each by the *γ*-probe. All lymph nodes accumulating activity were harvested and initially termed sentinel nodes. A definition of the true sentinel node was to be based on the results. After separate resection of the sentinel lymph nodes neck dissection was continued. It was carried out unilaterally in 39 patients and bilaterally in 21 patients. The extent of the neck dissection was depending on location and size of the primary tumour. In three patients with a tumour not crossing the midline sentinel nodes were observed on both sides of the neck. A bilateral neck dissection had been determined before the sentinel node procedure. Therefore no change in policy was necessary. If feasible, the primary tumour was excised before neck dissection in order to reduce the shine-through and scatter from the injection site, which can significantly hinder the detection of the sentinel lymph node. Location of sentinel lymph nodes, metastases and extent of neck dissection are described according to the terminology of the American Academy of Otolaryngology – Head and Neck Surgery (Robbins *et al*, 2002).

Neck dissection specimens and sentinel nodes were fixed in 10% neutral buffered formalin. For pathological examination, nodes were bisected along the axis. All nodes including sentinel lymph nodes were evaluated by a hematoxylin–eosin (H&E)-stained single section. If the single slices of the sentinel lymph nodes were free of tumour cells, they were further examined by step serial sections in 2 mm intervals in 18 patients. Of each block three sections were cut. One slice was stained with H&E and a second one immunohistochemically with cytokeratin antibody Lu5 (BMA, Augst, Switzerland). Results of the pathohistological examination of the sentinel lymph nodes and the respective neck dissection specimens were compared.

## RESULTS

In 46 out of 50 patients, there was a lymphatic drainage of the radiocolloid into at least one sentinel lymph node. In four patients, a sentinel lymph node could not be detected. Of 42 patients with a tumour not crossing the midline, 35 had an ipsilateral and three a bilateral drainage into sentinel lymph nodes while four did not reveal any sentinel nodes. Of eight patients with a tumour crossing the midline, seven had a bilateral and one a unilateral lymphatic drainage. The number of lymph nodes accumulating tracer and being biopsied intraoperatively varied from 1 to 11. The average number of lymph nodes accumulating the tracer was 3.2 (Table 1). There was no correlation between time interval between injection of the tracer and localisation of the sentinel nodes to the number of nodes detected.

Three of 11 patients with preoperative lymphoscintigraphy did not reveal a lymphatic drainage during scintigraphy, while in these three patients radiolabelled sentinel lymph nodes were detected intraoperatively with the *γ*-probe. In two of them an occult metastasis was found. In the remaining eight patients, a lymphatic drainage was observed by scintigraphy. The drainage into the levels of the neck was identical to the location of sentinel lymph nodes detected with the *γ*-probe. In six cases, however, there were more sentinel nodes detected with the probe than visualised by scintigraphy.

In 34 patients pathohistological examination did neither show occult metastases in the sentinel nodes nor in lymph nodes of the neck dissection specimens. In 12 patients at least one sentinel lymph node was found to harbour occult metastastic disease. In nine of these patients metastases in the sentinel lymph nodes were the only ones, whereas in three patients additional metastases were found in nonsentinel lymph nodes. Thus, the status of the neck had to be changed from N0 to pN1 in five patients, to pN2b in six patients and due to a contralateral metastasis to pN2c in one patient. All sentinel nodes containing occult metastases were within the first five nodes of highest activity in each patient (Table 2

**Table 2:** Background activity and count rate of radiolabelled sentinel lymph nodes

**No.**	**Background counts**	**1. SLN**	**2. SLN**	**3. SLN**	**4. SLN**	**5. SLN**	**6. SLN**	**7. SLN**	**8. SLN**	**9. SLN**	**10. SLN**	**11. SLN**
5	37	**3698**	2734	1368	750							
6	12	1590	1407	**1364**	665	519	404	403	346	274	265	221
13	40	**181**	160									
18	71	**990**	**578**									
19	17	**121**										
21	5	**1975**	204	142	91							
22	6	1250	945	**542**	**112**	98	76					
33	22	**744**										
37	18	192	**66**	51								
39	2	90	89	**50**	45	**37**	12					
40	4	**41**										

Lymph nodes marked in bold letters signify occult metastatic disease. SLN=sentinel lymph node.

). None of the patients with tumour-free sentinel lymph nodes revealed metastases in nonsentinel lymph nodes.

In three patients without detectable sentinel lymph nodes, no metastases were found by pathohistologic examination. In one patient without accumulation of the tracer in lymph nodes a single metastasis was detected in the neck dissection specimen.

In two of 12 patients, occult metastases were detected only after additional sections had been stained with H&E. In one patient, the metastasis was found by immunohistochemical staining only.

Of the four patients in whom a peritumoral injection was not possible and who received only part of the injection, two patients with laryngeal carcinomas were staged pN0 after pathohistologic examination. In the remaining two patients lymph nodes accumulating tracer were tumour-free whereas in each case a node without tracer accumulation harboured metastatic disease.

## DISCUSSION

The sentinel node concept offers the chance to stage the neck with less morbidity than an elective selective neck dissection. Attempts in assessing the diagnostic value of the sentinel node by fine-needle aspiration cytology offering the least possible morbidity, though at first promising, were not successful (Colnot *et al*, 2001; Höft *et al*, 2002; Nieuwenhuis *et al*, 2002). The method does not reliably detect occult metastatic disease as the sample of the sentinel lymph node is too small. Achieving a valid diagnosis mandates a pathohistological examination of a complete sentinel node. In order to determine the diagnostic value of the sentinel lymph-node concept, we compared the pathohistological results of sentinel nodes with the respective neck dissection specimens.

In our group of patients, the pathological exclusion of occult metastases in sentinel lymph nodes was predictive for the pathological status of the neck in each patient. Based on the limited number of patients in this study, the sentinel lymph node seems to have a high diagnostic value in head and neck cancer. This is in accordance with the literature. The first biopsy of a radiolabelled sentinel lymph node in head and neck cancer was performed by Alex and Krag (1996). Initial investigations of the sentinel lymph-node concept in head and neck cancer were disappointing using blue dye only (Alex and Krag, 1996; Pitman *et al*, 1998; Shoaib *et al*, 1999). Studies applying a radiolabelled tracer with or without additional blue dye, however, were promising. The majority of the studies reported results favouring the sentinel lymph-node concept (Koch *et al*, 1998; Shoaib *et al*, 1999, 2001; Alex *et al*, 2000; Zitsch *et al*, 2000; Mozzillo *et al*, 2001; Stoeckli *et al*, 2001, 2002; Barzan *et al*, 2002; Werner *et al*, 2002a, 2002b). Groups who worked with blue dye report of an extravasation of the blue dye into the tissue (Pitman *et al*, 2002). Other authors detected stained sentinel nodes only in a minority of their patients (Stoeckli *et al*, 2001). Additionally, blue dye will stain the area around the primary tumour. This hinders a resection of the primary tumour and might alter the absorption of the laser energy that is frequently used to resect oral, pharyngeal and laryngeal tumours. Therefore, most groups in contrast to groups treating malignant melanomas or breast cancer prefer a radioactive tracer without using blue dye.

We excluded four patients from our study in whom a peritumoral injection of the caudal rim of the tumour was not possible. Since the tracer had already been injected, nodes accumulating tracer were localised. Pathohistological comparison revealed occult metastatic disease in two of the patients. However, the nodes that had accumulated the tracer were free of metastases. Thus, if a complete peritumoral injection of the tracer is not possible, the patient is not eligible for the sentinel node method.

In four of our patients, no sentinel node could be detected. Reasons might be a wrong technique by injecting the tracer too deep into the tissue not close enough to the mucosa. Another reason might be that in-transit metastases diverted or in this case blocked the drainage of the radiocolloid into the sentinel node (Civantos *et al*, 2003).

In 10 of 13 patients with occult metastases, the initial H&E staining was sufficient to detect the metastases. In two of the patients, however, they were discovered exclusively after additional sections had been stained with H&E. In another case, tumour cells were found by immunohistochemical staining only. An intensive sectioning and standard H&E staining as well as immunohistochemical staining will reveal more metastases than standard single-block examination of a lymph node (Ambrosch *et al*, 1995; van den Brekel *et al*, 1996). Thus, performing a sentinel node-biopsy and basing the therapy of the neck on the status of the sentinel lymph node mandates an intensive and profound patho- and immunohistochemical work-up.

In one patient 10 and in another patient 11 radioactive lymph nodes were found. It is obvious that not all these nodes were sentinel lymph nodes. A high number of nodes accumulating activity poses a problem as the aim of the sentinel node concept is to keep the surgical morbidity to a minimum. Yet, also in other studies up to nine nodes accumulating tracer have been detected (Alex *et al*, 2000; Shoaib *et al*, 2001). There have been different approaches to limit the number of nodes to be biopsied by defining the sentinel lymph node by its activity. Alex *et al* (2000) suggested that an activity three times higher than the background should classify a sentinel lymph node. Zitsch *et al* (2000) adopted a definition used in malignant melanomas defining the sentinel node as having two times the background activity count rate. Werner *et al* (2002a) characterised sentinel lymph nodes as the three nodes with the highest activity emitting at least 10 times the background counts. As depicted in Table 2, all sentinel lymph nodes with occult metastases were among the five nodes with the highest activity. Yet, even if we had limited ourselves to harvesting the three nodes with the highest activity, we still would have detected all patients with occult metastatic disease. Thus, defining the sentinel nodes as the three nodes with the highest activity seems to be sufficient to reduce the number of nodes to be resected while achieving an oncologic sound result.

Counts of the background and of the sentinel nodes varied considerably. This might be due to individual differences, although the technique has been standardised as described. Depth of injection might differ slightly resulting in a reflux of the tracer out of the tissue causing a higher background from the pharynx. Also less tracer will reach the lymph nodes. Patients 39 and 40 had small tumours. Therefore, less than 50 MBq was injected. Consequently, this might result in a reduced count both of the sentinel nodes and of the background. No correlation of the varying counts in respect to time interval between injection and detection of the sentinel nodes was found. However, since sentinel nodes are defined as the three nodes with the highest activity and not in relation to the average counts of all patients differences to the average do not have an impact on the individual patient.

Preoperative lymphoscintigraphy did not improve the procedure of identifying the sentinel nodes: in two patients without scintigraphic drainage sentinel nodes containing occult metastases were localised by the *γ*-probe. In the other patients more radiolabelled nodes were detected intraoperatively by the *γ*-probe than visualised by scintigraphy. In these cases, scintigraphy did not reduce the number of nodes to be biopsied as all were located in the same levels of the neck as the nodes visualised preoperatively. Thus, there seems to be little help by preoperative lymphoscintigraphy and we do not apply it on a regular basis any longer. An argument in favour of preoperative lymphoscintigraphy is the existence of aberrant drainage patterns to the contralateral side of the neck. However, careful percutaneous scanning with the *γ*-probe should detect radiolabelled lymph nodes there, too.

The average incidence of occult metastatic disease was 26% based on staging by ultrasound and ultrasound-guided fine-needle aspiration cytology. These results are comparable to the findings of other groups also applying radiological criteria for staging (Stoeckli *et al*, 2001; Barzan *et al*, 2002; Werner *et al*, 2002a). The better the staging methods are in detecting small metastases, the less occult metastases will be overlooked and the more valuable will be the impact of an additional sentinel lymph-node procedure. Consequently, it is important not to replace standard staging methods by the sentinel lymph-node concept, but to perform it in addition to the best possible staging procedures.

In the present group, no patient with tumour-free sentinel nodes was found to have a metastasis in a nonsentinel lymph node. Therefore, one could argue that if we had performed a sentinel biopsy only, an elective neck dissection could have been avoided in 34 of our 50 patients. Yet, data are too limited to permit this step. So far, only Ross *et al* (2002) have reported on a study of a true biopsy of the sentinel lymph node without elective neck dissection. In case of occult metastatic disease, therapeutic neck dissection is performed. However, so far, there have been no sufficient follow-up data. A validation of the sentinel lymph-node method mandates that patients with a mere biopsy of the sentinel nodes should have equal regional control rates as patients after elective neck dissection. The consequence of a tumour-positive sentinel lymph node has to be discussed, too. A subsequent therapeutic neck dissection will be delayed by several days until sentinel nodes have been examined intensively by patho- and immunohistology. Revision neck dissection will cause additional morbidity. Alternatively, radiotherapy could be applied. This again would cause severe morbidity. Intraoperative examination of the sentinel nodes would enable an instant decision whether or not to perform a therapeutic approach to the neck and to avoid a second surgical step. Frozen section examination of the sentinel lymph nodes has been performed in breast cancer and malignant melanomas. However, sensitivity especially for micrometastases is low and therefore frozen section examination is not recommended in these tumours (Turner *et al*, 1999; Koopal *et al*, 2000). Likewise, Civantos *et al* (2003) discovered only six of 10 occult metastases of squamous cell carcinomas of the oral cavity by frozen section examination. Thus, at present, there are still a number of problems to be solved before sentinel lymph-node biopsy can be integrated into clinical routine. Further studies with a combined sentinel node biopsy and elective neck dissection will have to clarify whether or not early metastases can be detected by sentinel node biopsy only. If this can be proven, more studies will be necessary to determine whether or not regional control after sentinel biopsy is equivalent to elective neck dissection and whether or not sentinel node biopsy with a possible secondary therapeutic neck dissection results in less morbidity than a primary limited selective elective neck dissection.
